# Potential miRNA-gene interactions determining progression of various ATLL cancer subtypes after infection by HTLV-1 oncovirus

**DOI:** 10.1186/s12920-023-01492-0

**Published:** 2023-03-28

**Authors:** Mohadeseh Zarei Ghobadi, Elaheh Afsaneh, Rahman Emamzadeh, Mona Soroush

**Affiliations:** 1 Department of Cell and Molecular Biology and Microbiology, Faculty of Biological Science and Technology, University of Isfahan, Isfahan, Iran; 2 Independent Researcher, Tehran, Iran; 3 Institute of Biochemistry and Biophysics (IBB), University of Tehran, Tehran, Iran

**Keywords:** Asymptomatic carriers, ATLL subtypes, HTLV-1, Interaction, WGCNA

## Abstract

**Background:**

Adult T-cell Leukemia/Lymphoma (ATLL) is a rapidly progressing type of T-cell non-Hodgkin lymphoma that is developed after the infection by human T-cell leukemia virus type 1 (HTLV-1). It could be categorized into four major subtypes, acute, lymphoma, chronic, and smoldering. These different subtypes have some shared clinical manifestations, and there are no trustworthy biomarkers for diagnosis of them.

**Methods:**

We applied weighted-gene co-expression network analysis to find the potential gene and miRNA biomarkers for various ATLL subtypes. Afterward, we found reliable miRNA-gene interactions by identifying the experimentally validated-target genes of miRNAs.

**Results:**

The outcomes disclosed the interactions of miR-29b-2-5p and miR-342-3p with *LSAMP* in ATLL_acute, miR-575 with *UBN2*, miR-342-3p with *ZNF280B*, and miR-342-5p with *FOXRED2* in ATLL_chronic, miR-940 and miR-423-3p with C6orf141, miR-940 and miR-1225-3p with *CDCP1*, and miR-324-3p with *COL14A1* in ATLL_smoldering. These miRNA-gene interactions determine the molecular factors involved in the pathogenesis of each ATLL subtype and the unique ones could be considered biomarkers.

**Conclusion:**

The above-mentioned miRNAs-genes interactions are suggested as diagnostic biomarkers for different ATLL subtypes.

**Supplementary Information:**

The online version contains supplementary material available at 10.1186/s12920-023-01492-0.

## Background

Adult T-cell leukemia/lymphoma (ATLL) is virus-caused cancer that is developed after infection by Human T-cell leukemia virus type-1 (HTLV-1) [1]. ATLL is diagnosed by the aggressive T-cell and malignant lymphoproliferations which are increased in the infected individuals after likely a long latency period [2]. The prevalence of ATLL is approximately 5% among HTLV-1 infected cases. Based on Shimoyama classification, ATLL is categorized into four major subtypes: acute, lymphoma, chronic, and smoldering. The first two are aggressive with a poor prognosis and the last two include an indolent clinical period with disparate clinicopathologic characteristics. The acute type is more common and usually is associated with high amounts of serum lactose dehydrogenase and leukemia. The lymphoma cells are present in the blood and affect the bones, skin, lymph nodes, spleen, and liver. In addition, lymphomatous ATLL is infrequent and grows quickly. Also, it can impress the brain and spinal cord with an increase in the lymph nodes. Chronic ATLL develops leisurely similar to the smoldering type and elevates T cells and lymphocytes in the blood. It can influence the lungs, skin, spleen, liver, and lymph nodes. Smoldering ATLL can also affect the lungs and skin which leads to unusual T-cell counts [3–5].

MicroRNAs (miRNAs) are a category of non-coding RNAs with a length of almost 19–25 nucleotides that regulate the expression of different genes. They have effects on various biological functions such as proliferation, cell cycle, apoptosis, differentiation, and immune response. The conceivable roles of miRNAs in the progression of ATLL and tumorigenesis have been specified [6–8].

Different ATLL subtypes have a poor prognosis because of the intrinsic chemoresistance and the severe immunosuppression in addition to their heterogeneous advent. The combination of chemotherapy drugs and miRNAs can be a suitable remedy for ATLL [9]. Several papers have introduced the genes and miRNAs implicated in the progression of ATLL without considering different subtypes [10–12]. Therefore, the exploration of miRNA-gene interactions in various ATLL subtypes to propose potential therapeutic targets using computational algorithms could be advantageous.

Weighted gene co-expression network analysis (WGCNA) is a potent algorithm that could cluster the genes through the calculation of correlations between them. The identified clusters named modules contain the co-expressed gene groups which likely participate in the same biological pathways. Moreover, assessing the preservation of the identified modules in the external data could lead to identifying the specific modules involved in disease [10].

We recently used machine learning to classify different ATLL subtypes based on the mRNA and miRNA datasets [9]. However, we could only find one common miRNA and a few genes for each subtype. In this study, we employed the weighted gene co-expression method for finding specific coding and non-coding RNA interactions for three subtypes of ATLL. It sheds light on the pathogenesis mechanisms from asymptomatic carriers (ACs) toward the progression of each ATLL subtype.

## Materials and Methods

### Gene expression datasets and preprocessing

The microarray gene expression datasets GSE33615 [13], GSE55851 [14], GSE29312 [15], and GSE29332 [15] were downloaded from the database Gene Expression Omnibus (GEO). The two first datasets include the gene expression levels in the Peripheral Blood Mononuclear Cells (PBMCs) or the whole blood of patients with one of the ATLL subtypes including acute, chronic, and smoldering. The last two datasets contain the gene expression levels in the PBMCs of AC carrier samples. Totally, 29, 23, and 10 subjects including ATLL with acute, chronic, and smoldering subtypes, respectively, as well as 37 AC subjects were used for further analysis. In addition, GSE31629 [13] and GSE46345 [16] datasets containing the miRNAs expression levels of 40 ATLL and 12 ACs subjects were employed to analyze the non-coding RNA data. The dataset details are explained in Table 1. The possible batch effect among datasets was removed using the function of removeBatchEffect in the Limma package version 3.54 in the R 4.2.2 environment [10, 17–21]. The data was also quantile normalized.

**Table 1:** Details of the datasets involved in the analysis

Dataset	Number of Samples	Link to dataset
**Gene datasets**		
GSE33615	Acute: 26 Chronic: 20 Smouldering: 4	https://www.ncbi.nlm.nih.gov/geo/query/acc.cgi?acc=GSE33615
GSE55851	Acute: 3 Chronic: 3 Smouldering: 6	https://www.ncbi.nlm.nih.gov/geo/query/acc.cgi?acc=GSE55851
GSE29312	ACs: 20	https://www.ncbi.nlm.nih.gov/geo/query/acc.cgi?acc=GSE29312
GSE29332	ACs: 17	https://www.ncbi.nlm.nih.gov/geo/query/acc.cgi?acc=GSE29332
**miRNA datasets**		
GSE31629	ATLL: 40	https://www.ncbi.nlm.nih.gov/geo/query/acc.cgi?acc=GSE31629
GSE46345	ACs: 12	https://www.ncbi.nlm.nih.gov/geo/query/acc.cgi?acc=46345

### Weighted gene co-expression network

The weighted gene co-expression network was constructed employing the R package “WGCNA” version 1.71 [22]. WGCNA was used to find clusters of co-expressed genes that likely are involved in similar biological pathways. To identify these clusters, known as modules, an adjacency matrix was initially calculated using Pearson correlation between pairs of genes/miRNAs, with the optimized soft power. The “pickSoftThreshold” function was used to identify scale-free topology fitting indices against different soft thresholding powers β. Afterward, the Topological Overlap Matrix (TOM) was determined by transforming the adjacency matrix. Highly co-expressed genes were then grouped using hierarchical clustering. Next, the dynamic tree cut algorithm was applied to cut dendrogram branches and to identify gene modules. The close modules were merged utilizing the mergeCloseModules function.

### Identification of specific modules for each subtype

In this step, the module’s preservation for each individual ATLL subtype in the ACs expression dataset was determined. To this end, the “modulePreservation” function in the WGCNA package (version 1.71) was utilized. The module preservation statistics introduced a measure indicating the preservation or somewhat non-preservation of a module between a reference network and a test network [23]. In this study, the co-expression networks of ATLL subtypes were considered as the reference and ACs as the test network. The same analysis was performed for the miRNA dataset. The parameters of *Z*_*summary*_ ($\frac{ {Z}_{\text{d}\text{e}\text{n}\text{s}\text{i}\text{t}\text{y}}+ {Z}_{\text{c}\text{o}\text{n}\text{n}\text{e}\text{c}\text{t}\text{i}\text{v}\text{i}\text{t}\text{y}}}{2}$) and *medianRank* ($\frac{ {\text{m}\text{e}\text{d}\text{i}\text{a}\text{n}\text{R}\text{a}\text{n}\text{k}}_{\text{d}\text{e}\text{n}\text{s}\text{i}\text{t}\text{y}}+ {\text{m}\text{e}\text{d}\text{i}\text{a}\text{n}\text{R}\text{a}\text{n}\text{k}}_{\text{c}\text{o}\text{n}\text{n}\text{e}\text{c}\text{t}\text{i}\text{v}\text{i}\text{t}\text{y}}}{2}$) were measured to determine the preservation of modules. *Z*_*summary*_ and *medianRank* combine various preservation statistics into individual measures of preservation. These two measures are both important for deciding the preservation of a network module. In this study, *Z*_*summary*_ determines whether modules identified in the ATLL datasets remain highly connected in the ACs dataset (density) and whether the connections between the genes in each module are the same between the ATLL and ACS datasets (connectivity) [24]. The *medianRank* is beneficial to compare the preservation among several modules so that a module with a higher *medianRank* shows weaker preservation statistics than a module with a lower median rank. It is highly independent of module size [23]. Modules with *Z*_*summary*_<2 and *medianRank*≥8 were regarded as non-preserved gene co-expression modules in the ACs group and so are specific for each ATLL subtype [25–27]. Moreover, *Z*_*summary*_<2 was considered to determine specific miRNA co-expression modules for ATLL.

### Deteremining differentially expressed genes and miRNAs

To determine the differentially expressed genes (DEGs) and differentially expressed miRNAs (DEMs) between ATLL and ACs groups, the Bioconductor package Limma (version 3.54) was employed. The statistically meaningful DEGs and DEMs were identified by applying Benjamini-Hochberg adjusted *p*-value [28] cutoff of less than 0.05.

### Identification of target genes for miRNAs

The unique DEGs in the preserved modules in each ATLL subtype were determined (U_DEGs). Moreover, the unparalleled DEMs in the preserved modules in ATLL were also found (U_DEMs). Next, the miRTarBase database containing the experimentally validated miRNA-target gene interactions was searched to determine the target genes of the U_DEMs [8]. Afterward, the common genes between these target genes and U_DEGs were determined (C_DEGs). Finally, the interactions of miRNA-genes was depicted in Cytoscape 3.6.1.

### Stepwise method to perform analysis

**Fig. 1:**
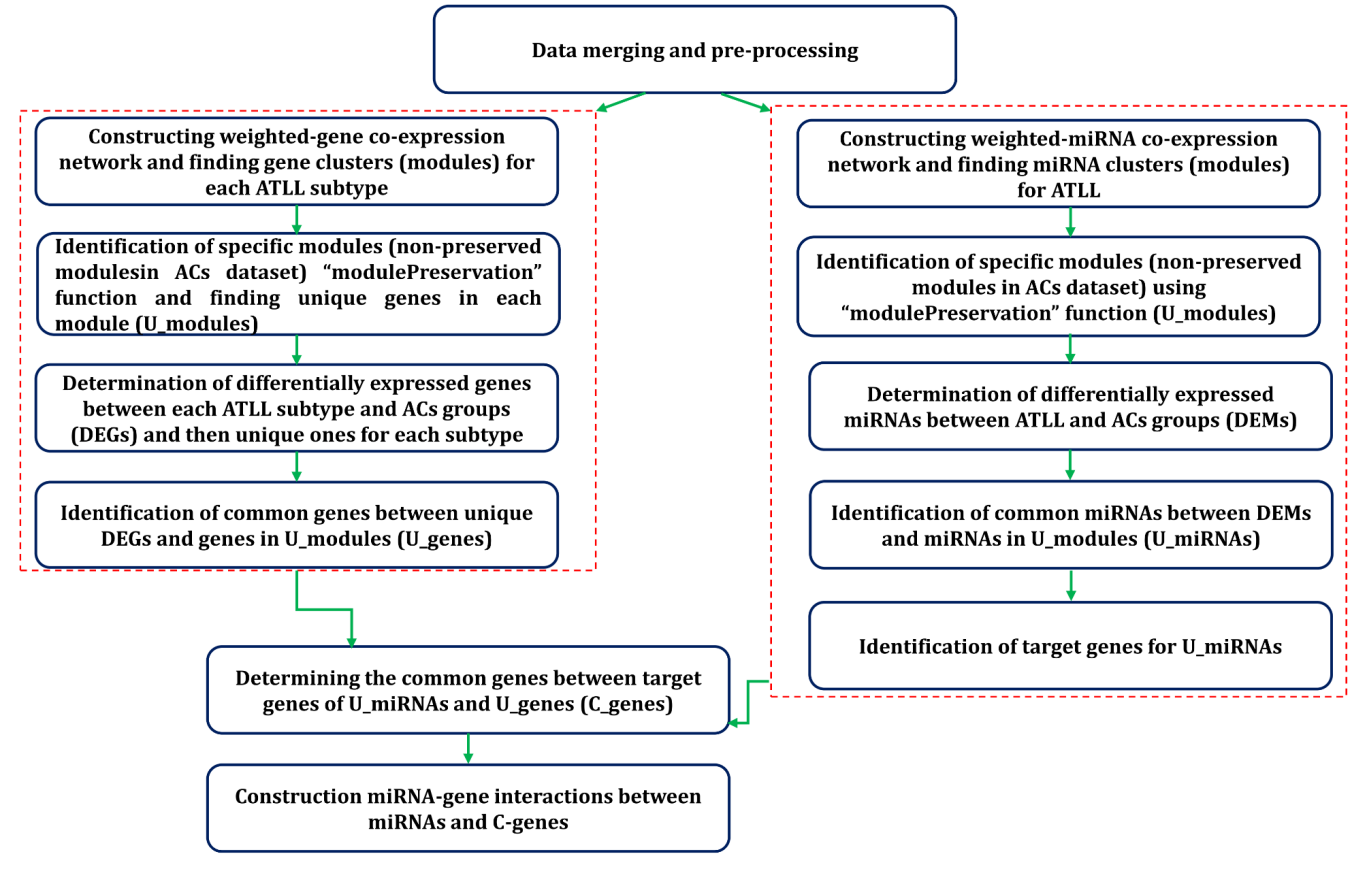
Flowchart of the step-wise analyses in this study

The steps of the performed analyses in this study are shown in a flowchart (Fig. 1). Briefly, we first prepared data for further analysis by merging different datasets and pre-precessing. Then, we constructed the weighted gene/miRNAs co-expression networks. Afterward, we determined the specific gene modules for each ATLL subtype/miRNA module for ATLL through performing module preservation analysis and finding unique genes in each gene module (U_modules). In the next step, we identified DEGs and DEMs between ATLL and ACs and then found unique DEGs for each subtype. We further identified shared genes between unique DEGs and genes in U_modules (U_genes) as well as common miRNAs between DEMs and miRNAs in U_modules (U_miRNAs). Following the determination of the target genes of U_miRNAs, we explored the shared genes between the target genes of U_miRNAs and U_genes (C_genes). Finally, we constructed miRNA-gene interactions between miRNAs and C-genes for each subtype.

## Results

### Construction of WGCNs

**Fig. 2:**
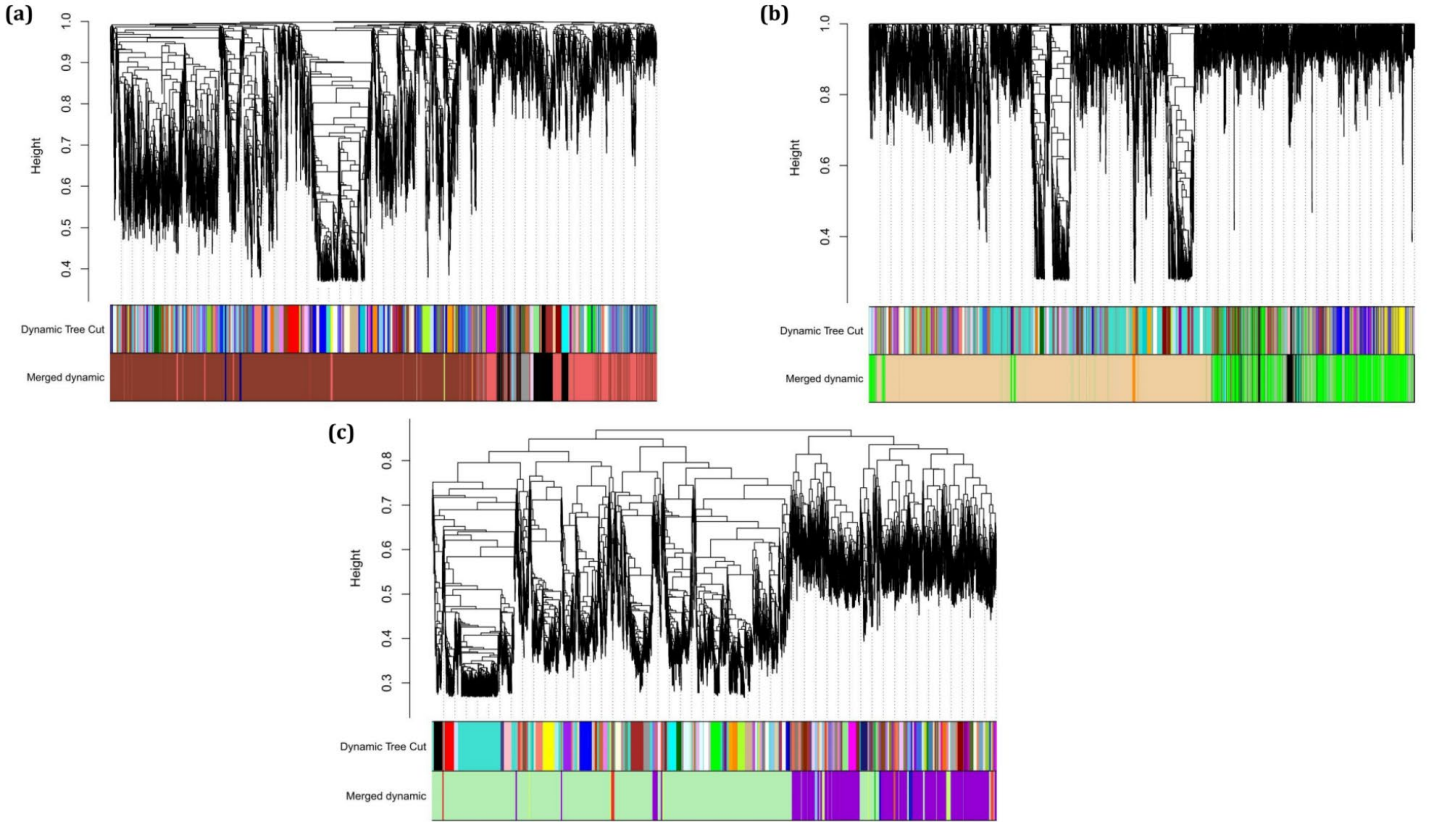
Dendrogram of clustered genes constructed by WGCNA based on (1-TOM) for (a) ATLL acute subtype (ATLL_acute), (b) ATLL chronic subtype (ATLL_chronic), and (c) ATLL smoldering subtype (ATLL_smoldering) with the specified module colors. Each color denotes a module (group of genes) determined by the dynamic tree cut algorithm before and after merging modules

**Fig. 3:**
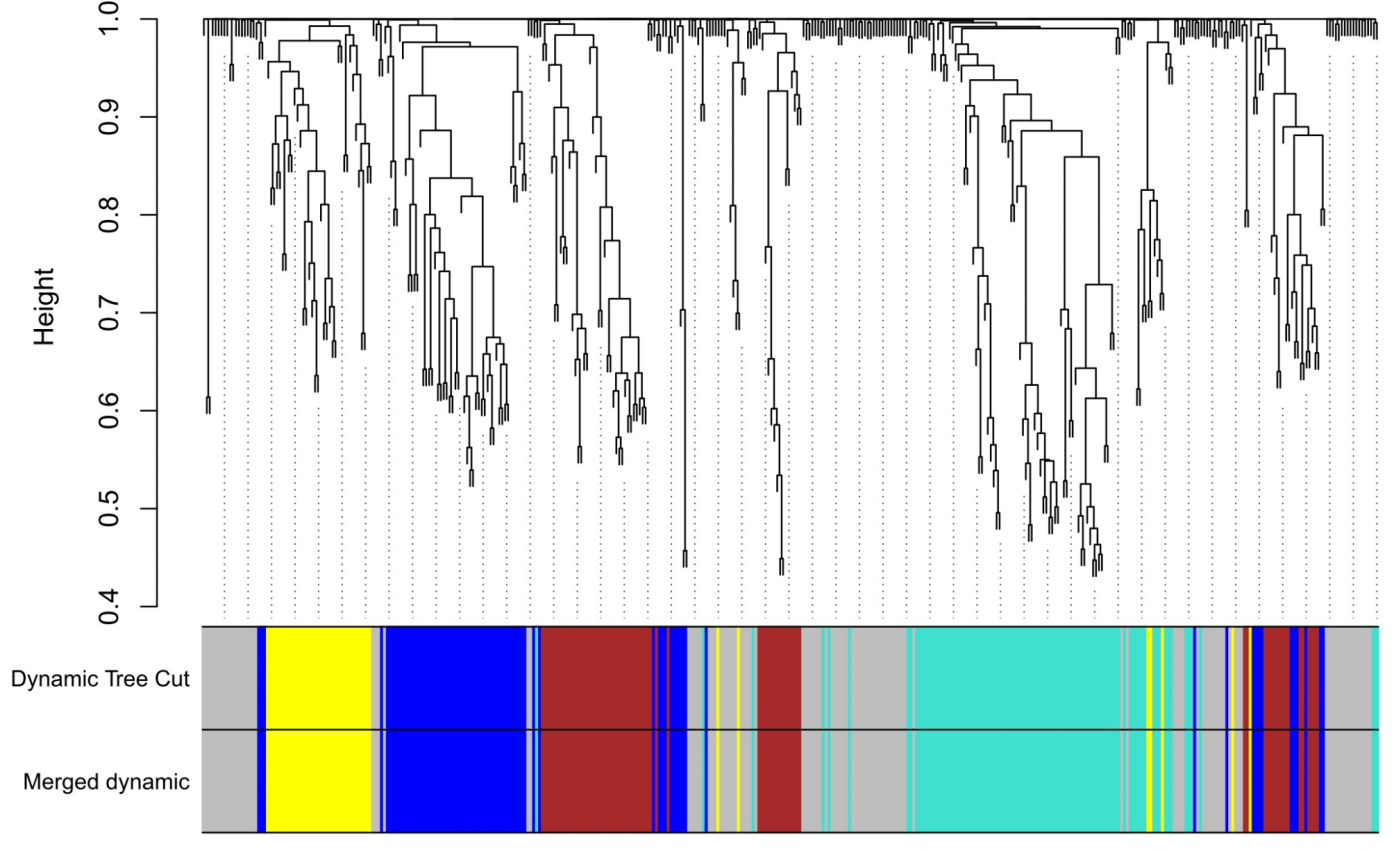
Dendrogram of clustered genes constructed by WGCNA based on (1-TOM) for miRNA dataset of ATLL with the specified module colors. Each color denotes a module (group of genes) determined by the dynamic tree cut algorithm before and after merging modules

A total of 14,837 common genes were used to construct three weighted co-expression networks for three ATLL subtypes. At first, the soft-thresholding power (β) of 7, 17, and 2 were determined as the optimum quantities to obtain a scale-free topology for acute, chronic, and smoldering, respectively. After calculating adjacency matrix power β, TOM dissimilarity, hierarchical clustering, cutting the clusters, and finally merging the close clusters, nine modules were identified for ATLL_acute, seven modules for ATLL_chronic, and nine modules for ATLL_smoldering (Grey module contains the genes that are not assigned to any of the modules). Figure 2a-c indicates the dendrogram and the identified modules specified by a unique color for each subtype. Moreover, a weighted gene co-expression network was constructed for miRNA ATLL samples. No dataset comprising the miRNA expression for each ATLL subtype is available, so we presumed the miRNA expression for ATLL regardless of its subtype. The β of 10 was determined as the optimum value to reach a scale-free topology. Figure 3 demonstrates the dendrogram and the four obtained modules.

### Identification of non-preserved modules

**Fig. 4:**
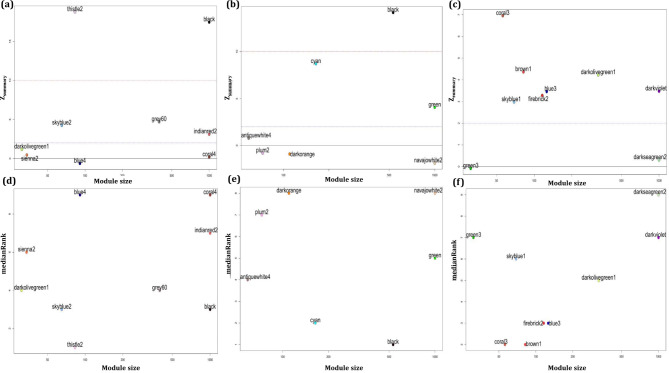
Preservation *Z*_*summary*_ (a-c) and *medianRank* (d-e) versus module size for ATLL acute subtype (ATLL_acute), ATLL chronic subtype (ATLL_chronic), and ATLL smoldering subtype (ATLL_smoldering), respectively. The modules below the dashed line ( *Z*_*summary*_<2 and medianRank ≥ 8) are the specific modules for each ATLL subtype

**Fig. 5:**
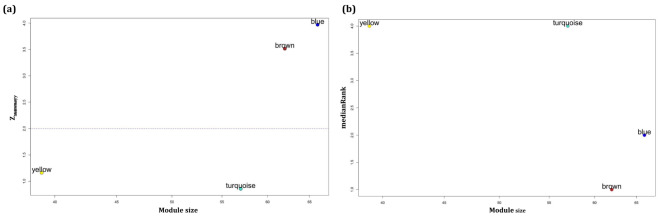
Preservation (a) *Z*_*summary*_ and (b) *medianRank* versus module size after constructing a weighted miRNA co-expression network. The modules below the dashed line ( *Z*_*summary*_<2 and *medianRank* ≥ 8) are the specific modules for ATLL.

To identify specific modules for each of the three ATLL subtypes, their preservations in the ACs dataset were investigated. The modules with *medianRank* ≥ 8 and *Z*_*summary*_ < 2 were considered as specific non-preserved gene modules and *Z*_*summary*_ < 2 for miRNA modules. Figure 4a-c demonstrates the plots of *Z*_*summary*_ scores and Fig. 4d-f indicates the plots of *medianRank* scores versus module size for ATLL_acute, ATLL_chronic, and ATLL_smoldering, respectively (Supplementary data file 1). Therefore, blue4 and coral4 modules in ATLL_acute, darkorange and navajowhite2 modules in ATLL_chronic, and darkseagreen2 module in ATLL_smoldering were found as specific and subtype-related modules. Figure 5a,b also represents the plots of *Z*_*summary*_ and *medianRank* scores for ATLL_miRNA and shows the preservation of turquoise and yellow modules in ATLL (Supplementary data file 1). Next, we determined the unique genes in each specific module among all ATLL subtypes. Since they are not present in any other modules, we referred to them as unique modules (U_modules, Supplementary data file 2). The miRNAs in the preserved modules in ATLL (turquoise and yellow) were also considered U_modules. In the further step, we determined DEGs between each ATLL subtype and ACs samples as well as DEMs between ATLL and ACs samples considering adj. *p*. value < 0.05. Then, the unique DEGs for each subtype were identified (Supplementary data file 3). Afterward, the common ones between genes/miRNAs in each U_module and DEGs/DEMs called U_genes/U_miRNAs were found (Supplementary data file 4).

### Constructing miRNA‑gene interactions

**Fig. 6:**
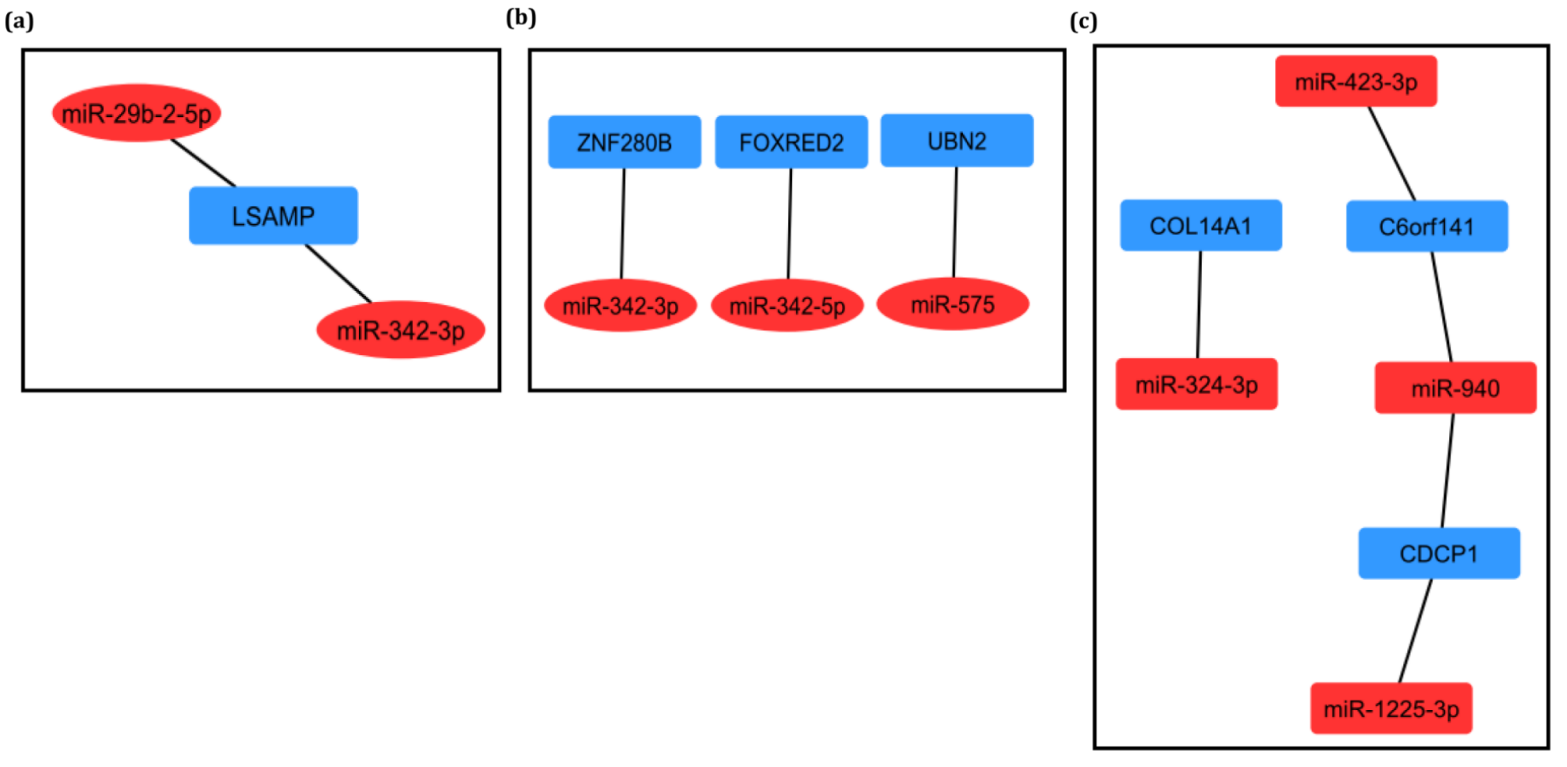
The unique miRNA-gene interactions for (a) ATLL_acute, (b) ATLL_chronic, (c) ATLL_smoldering.

To find the experimentally validated target genes of U_miRNAs, the miRTarBase database was explored (Supplementary data file 5). Next, the shared genes between the target genes and U_genes (C_genes) for each subtype were explored. As a result, the interactions of miR-29b-2-5p and miR-342-3p with *LSAMP* in ATLL_acute, miR-342-5p with *FOXRED2*, miR-342-3p with *ZNF280B*, and miR-575 with *UBN2* in ATLL_chronic, miR-1225-3p and miR-940 with CDCP1, miR-423-3p and miR-940 with C6orf141, miR-324-3p with COL14A1 in ATLL_smoldering were found (Fig. 6). The identified miRNA-gene interactions may be involved in the pathogenesis mechanism and development of each subtype. Moreover, the unique ones in these interactions could be considered potential biomarkers.

## Discussion

The identification of the potential role of genes and miRNAs in the development of each ATLL subtype is crucial for understanding the pathogenesis mechanism and identifying therapeutic targets. In this study, we utilized the weighted gene co-expression analysis procedure to identify the particular co-expressed genes in three subtypes of ATLL. In the following, we discuss the determined genes and miRNAs that probably have the main roles in the progression of each ATLL subtype cancer.

In the acute subtype, *LSAMP* gene and its interaction with miR-29b-2-5p and miR-342-3p were identified. LSAMP encodes a neuronal surface glycoprotein present in the subcortical and cortical regions of the limbic system. LSAMP can be involved in tumor suppression and neuropsychiatric disorders [29, 30]. Furthermore, miR-29b-2-5p and miR-342-3p barricade cell proliferation and promote apoptosis. Their functions have been determined in several cancers, such as pancreatic ductal adenocarcinoma, cervical cancer, and non-small cell lung cancer [31–33]. The lower expression of *LAMP* may be related to the higher expressions of miR-29b-2-5p and miR-342-3p that ultimately result in tumor suppression [30].

In the chronic subtype of ATLL, FOXRED2 and ZNF280B were found to have interconnections with miR-342-5p and miR-342-3p, respectively, and UBN2 was also identified to have an interaction with miR-575. FOXRED2 is an unstable protein that is probably implicated in the ubiquitin-dependent ERAD pathway and is essential for the modulation of the proteasome [34]. The inhibitors of proteasome induce apoptosis, which can have an antitumor effect [35]. The function of FOXRED2 in cancer is not yet fully understood, and further studies are required to investigate its role in chronic ATLL. *ZNF280B* is known as an oncogene that encodes a transcription factor protein inducing the overexpression of *MDM2*. MDM2 boosts tumor constitution and cancer cell growth by targeting some tumor repressor proteins like p53 [36, 37]. MiR-342-5p is a downstream molecule of Notch signaling implicated in the regulation of Endothelial cells (ECs) during angiogenesis. Its higher expression weakens angiogenesis and promulgated EndMT. MiR-342-5p likely acts as a tumor suppressor and may also suppress migration and cell proliferation [38, 39]. Similarly, miR-342-3p represses cell growth and proliferation and also inhibits migration and invasion [32, 40]. The overexpression of these two miRNAs by targeting *ZNF280B* and *FOXRED2* could suppress tumorigenesis and cell proliferation in chronic ATLL.

On the other hand, UBN2 is a nuclear protein with the capability of interacting with several transcription factors. It acts as an oncogene that can be involved in the proliferation and tumorigenicity of cancer cells [41]. UBN2 can contribute to the transcription of the KRAS gene as a sector of histone chaperone. The cell cycle can be regulated by KRAS signaling through phosphorylation and interdicting p21 and p27 to mitigate cyclinD1 [42]. UBN2 is targeted by miR-575 as an oncomir that can boost cell proliferation and migration in some cancer cells and possibly chronic ATLL [43–45].

In the smoldering subtype of ATLL, *CDCP1*, *C6orf141*, and *COL14A1* were found. CDCP1 is a known protein implicated in malignancies of multiple cancers. It associates with important tumorigenic signaling cascades, comprising the PI3K/AKT, SRC/PKCδ, RAS/ERK, WNT axes, and oxidative pentose phosphate pathway [46]. Therefore, CDCP1 is a considerable therapeutic and diagnostic target [47]. C6orf141 has been found as a tumor repressor protein in oral cancer. Its promoter CpG islands are methylated in some cancer which communicates with high-density lipoprotein alterations [48]. *COL14A1* is another gene whose role has not been fully understood in cancers. It is methylated in renal cell carcinoma that may act as a tumor suppressor. It associates with a poorer prognosis independent of tumor grade, size, and stage [46]. Also, it has been identified that COL14A1 has an important role in keeping the stem cell-like and self-renewal features of Liver cancer stem cells through the activation of ERK signaling [47]. MiR-940 interdicts proliferation and migration of cancer cells and miR-1225-3p implicates malignancy. These two miRNAs interact with CDCP1 [48, 49]. Moreover, miR-423-3p is an oncomir that boosts cancer cell proliferation through the promotion of the G1/S transition phase of the cell cycle [50, 51]. It is in association with miR-940 target C6orf141 in smoldering ATLL. On the other hand, miR-324-3p which targets *COL14A1*, suppresses the invasion and growth of some cancer cells by elevating the apoptosis [52]. Also, it was proposed that the miR-324-3p/Smad4/Wnt signaling axis could be a therapeutic target to barricade cancer progression [53]. However, more studies must be performed for finding its convenient role in tumorigenesis.

On the whole, the miRNA-gene interaction networks that may contribute to the pathogenesis of each ATLL subtype were proposed. However, these networks represent only a small fraction of the complex network involved in ATLL development, and additional data are required to unveil the complete network. Therefore, future studies with larger cohorts are necessary to determine the comprehensive interaction of genes and miRNAs in each ATLL subtype.

## Conclusion

In summary, we found the genes and miRNAs that could be significantly involved in the pathogenesis of three ATLL subtypes. The step-wise analysis revealed unique genes/miRNA in the identified interactions, including *LSAMP* and miR-29b-2-5p in acute, *FOXRED2*, *UBN2*, miR-342-5p, and miR-575 in chronic, and *CDCP*, *C6orf141*, *COL14A1*, miR-1225-3p, miR-940, miR-423-3p, miR-324-3p in smoldering subtypes. These genes and miRNAs could serve as potential biomarkers. However, their efficacies should be confirmed through experimental studies.

## Electronic supplementary material

Below is the link to the electronic supplementary material.

Supplementary Material 1

Supplementary Material 2

Supplementary Material 3

Supplementary Material 4

Supplementary Material 5

Supplementary Material 6

## Data Availability

The datasets generated and/or analysed during the current study are available in the Gene Expression Omnibus (GEO) repository [GSE33615, GSE55851, GSE29312, GSE29332, GSE31629, and GSE46345]. All data generated or analysed during this study are included in this article.

## References

[1] Zarei Ghobadi M, Emamzadeh R, Teymoori-Rad M, Mozhgani S-H (2021). Decoding pathogenesis factors involved in the progression of ATLL or HAM/TSP after infection by HTLV-1 through a systems virology study. Virol J.

[2] Futsch N, Prates G, Mahieux R, Casseb J, Dutartre H (2018). Cytokine networks dysregulation during HTLV-1 infection and associated diseases. Viruses.

[3] Malpica L, Pimentel A, Reis IM, Gotuzzo E, Lekakis L, Komanduri K, Harrington T, Barber GN, Ramos JC (2018). Epidemiology, clinical features, and outcome of HTLV-1–related ATLL in an area of prevalence in the United States. Blood Adv.

[4] Qayyum S, Choi JK (2014). Adult T-cell leukemia/lymphoma. Archives of Pathology Laboratory Medicine.

[5] Jabbour M, Tuncer H, Castillo J, Butera J, Roy T, Pojani J, Al-Malki M, Al-Homsi A (2011). Hematopoietic SCT for adult T-cell leukemia/lymphoma: a review. Bone Marrow Transplant.

[6] Moles R, Nicot C (2015). The emerging role of miRNAs in HTLV-1 infection and ATLL pathogenesis. Viruses.

[7] Ye F (2018). MicroRNA expression and activity in T-cell acute lymphoblastic leukemia. OncoTargets.

[8] Ghobadi MZ, Emamzadeh R, Mozhgani S-H (2021). Deciphering microRNA-mRNA regulatory network in adult T-cell leukemia/lymphoma; the battle between oncogenes and anti-oncogenes. PLoS ONE.

[9] Ghobadi MZ, Emamzadeh R, Afsaneh E (2022). Exploration of mRNAs and miRNA classifiers for various ATLL cancer subtypes using machine learning. BMC Cancer.

[10] Zarei Ghobadi M, Emamzadeh R (2022). Integration of gene co-expression analysis and multi-class SVM specifies the functional players involved in determining the fate of HTLV-1 infection toward the development of cancer (ATLL) or neurological disorder (HAM/TSP). PLoS ONE.

[11] Mozhgani S-H, Ghobadi MZ, Norouzi M, Rahimi H, Valizadeh N, Teymoori-Rad M, Tarokhian H, Ostadali M, Farajifard H, Rezaee SA (2022). Signaling factors potentially associated to the pathogenesis of adult T-cell leukemia/lymphoma: a network-analysis and novel findings assessment. Virus Res.

[12] Shadabi S, Delrish N, Norouzi M, Ehteshami M, Habibian-Sezavar F, Pourrezaei S, Madihi M, Ostadali M, Akhgar F, Shayeghpour A (2021). Comprehensive high-throughput meta-analysis of differentially expressed microRNAs in transcriptomic datasets reveals significant disruption of MAPK/JNK signal transduction pathway in adult T-cell leukemia/lymphoma. Infect Agents Cancer.

[13] Yamagishi M, Nakano K, Miyake A, Yamochi T, Kagami Y, Tsutsumi A, Matsuda Y, Sato-Otsubo A, Muto S, Utsunomiya A (2012). Polycomb-mediated loss of miR-31 activates NIK-dependent NF-κB pathway in adult T cell leukemia and other cancers. Cancer Cell Int.

[14] Fujikawa D, Nakagawa S, Hori M, Kurokawa N, Soejima A, Nakano K, Yamochi T, Nakashima M, Kobayashi S, Tanaka Y (2016). Polycomb-dependent epigenetic landscape in adult T-cell leukemia. Blood The Journal of the American Society of Hematology.

[15] Tattermusch S, Skinner JA, Chaussabel D, Banchereau J, Berry MP, McNab FW, O’Garra A, Taylor GP, Bangham CR (2012). Systems biology approaches reveal a specific interferon-inducible signature in HTLV-1 associated myelopathy. PLoS Pathog.

[16] Vernin C, Thenoz M, Pinatel C, Gessain A, Gout O, Delfau-Larue M-H, Nazaret N, Legras-Lachuer C, Wattel E, Mortreux F (2014). HTLV-1 bZIP factor HBZ promotes cell proliferation and genetic instability by activating OncomiRs. Cancer Res.

[17] Liu J, Liu S, Yu Z, Qiu X, Jiang R, Li W (2022). Uncovering the gene regulatory network of type 2 diabetes through multi-omic data integration. J Translational Med.

[18] Pan Z, Xu T, Bao L, Hu X, Jin T, Chen J, Chen J, Qian Y, Lu X, li L (2022). CREB3L1 promotes tumor growth and metastasis of anaplastic thyroid carcinoma by remodeling the tumor microenvironment. Mol Cancer.

[19] Wang T, Zeng F, Li X, Wei Y, Wang D, Zhang W, Xie H, Wei L, Xiong S, Liu CJCR. Identification of key genes and pathways associated with sex differences in rheumatoid arthritis based on bioinformatics analysis. 2022:1–8. 10.1007/s10067-022-06387-6 36173499

[20] He S, Deng Z, Li Z, Gao W, Zeng D, Shi Y, Zhao N, Xu F, Li T, Li H (2021). Signatures of 4 autophagy-related genes as diagnostic markers of MDD and their correlation with immune infiltration. J Affect Disord.

[21] De Silva K, Demmer RT, Jönsson D, Mousa A, Forbes A, Enticott J (2022). Highly perturbed genes and hub genes associated with type 2 diabetes in different tissues of adult humans: a bioinformatics analytic workflow. Funct Integr Genomics.

[22] Langfelder P, Horvath S (2008). WGCNA: an R package for weighted correlation network analysis. BMC Bioinformatics.

[23] Langfelder P, Luo R, Oldham MC, Horvath S (2011). Is my network module preserved and reproducible?. PLoS Comput Biol.

[24] Bakhtiarizadeh MR, Hosseinpour B, Shahhoseini M, Korte A, Gifani P (2018). Weighted gene co-expression network analysis of endometriosis and identification of functional modules associated with its main hallmarks. Front Genet.

[25] Salih SJ, Ghobadi MZ (2022). Evaluating the cytotoxicity and pathogenicity of multi-walled carbon nanotube through weighted gene co-expression network analysis: a nanotoxicogenomics study. BMC Genomic Data.

[26] Zarei Ghobadi M, Mozhgani S-H, Erfani Y. Identification of dysregulated pathways underlying HTLV-1-associated myelopathy/tropical spastic paraparesis through co-expression network analysis.Journal of neurovirology2021:1–11. 10.1007/s13365-020-00919-z 33405203

[27] Ghobadi MZ, Emamzadeh R, Teymoori-Rad M, Afsaneh E. Exploration of blood – derived coding and non-coding RNA diagnostic immunological panels for COVID-19 through a co-expressed-based machine learning procedure.Frontiers in Immunology2022,13. 10.3389/fimmu.2022.1001070 PMC9670818 36405703

[28] Benjamini Y, Hochberg Y (1995). Controlling the false discovery rate: a practical and powerful approach to multiple testing. J Roy Stat Soc B.

[29] Kresse SH, Ohnstad HO, Paulsen EB, Bjerkehagen B, Szuhai K, Serra M, Schaefer KL, Myklebost O, Meza-Zepeda LA (2009). Cancer: LSAMP, a novel candidate tumor suppressor gene in human osteosarcomas, identified by array comparative genomic hybridization. Genes Chromosomes.

[30] Chang C-Y, Wu K-L, Chang Y-Y, Liu Y-W, Huang Y-C, Jian S-F, Lin Y-S, Tsai P-H, Hung J-Y, Tsai Y-M (2021). The downregulation of LSAMP expression promotes lung cancer progression and is associated with poor survival prognosis. J Personalized Med.

[31] Li C, Dong Q, Che X, Xu L, Li Z, Fan Y, Hou K, Wang S, Qu J, Xu L (2018). MicroRNA-29b-2-5p inhibits cell proliferation by directly targeting Cbl-b in pancreatic ductal adenocarcinoma. BMC Cancer.

[32] Li X-r, Chu H-j, Lv T, Wang L, Kong S-f (2014). Dai S-z: mir-342-3p suppresses proliferation, migration and invasion by targeting FOXM1 in human cervical cancer. FEBS Lett.

[33] Xue X, Fei X, Hou W, Zhang Y, Liu L, Hu R (2018). Mir-342-3p suppresses cell proliferation and migration by targeting AGR2 in non-small cell lung cancer. Cancer Lett.

[34] Shim S, Lee W, Chung H, Jung Y-K (2011). Amyloid β-induced FOXRED2 mediates neuronal cell death via inhibition of proteasome activity. Cell Mol Life Sci.

[35] Voorhees PM, Dees EC, O’Neil B, Orlowski RZ (2003). The proteasome as a target for cancer therapy. Clin Cancer Res.

[36] Zhai J, Yang Z, Cai X, Yao G, An Y, Wang W, Fan Y, Zeng C, Liu K (2018). ZNF280B promotes the growth of gastric cancer in vitro and in vivo. Oncol Lett.

[37] Gao S, Hsieh C-L, Zhou J, Shemshedini L (2013). Zinc finger 280B regulates sGCα1 and p53 in prostate cancer cells. PLoS ONE.

[38] Yan XC, Cao J, Liang L, Wang L, Gao F, Yang ZY, Duan JL, Chang TF, Deng SM, Liu Y (2016). Mir-342‐5p is a notch downstream molecule and regulates multiple angiogenic pathways including notch, vascular endothelial growth factor and transforming growth factor β signaling. J Am Heart Association.

[39] Yang H, Li Q, Niu J, Li B, Jiang D, Wan Z, Yang Q, Jiang F, Wei P, Bai S. microRNA-342-5p and miR-608 inhibit colon cancer tumorigenesis by targeting NAA10. *OncoTargets* 2016, 7(3):2709. 10.18632/oncotarget.6458 PMC4823066 26646451

[40] Xie X, Liu H, Wang M, Ding F, Xiao H, Hu F, Hu R, Mei J (2015). Mir-342-3p targets RAP2B to suppress proliferation and invasion of non-small cell lung cancer cells. Tumor Biology.

[41] Zhao Y-l, Zhang ZS-R, Bi S-H, Xiao J-X, Wang Z-Y, Jiao S-Y, Zhang H-L, Qiu D, Zhang J-F (2019). UBN2 promotes tumor progression via the Ras/MAPK pathway and predicts poor prognosis in colorectal cancer. Cancer Cell Int.

[42] Le TT-H, Hsieh C-L, Lin I-H, Chu C-Y, Do AD, Chen S-H, Shigemura K, Kitagawa K, Fujisawa M, Liu M-C (2022). The ADAM9/UBN2/AKR1C3 axis promotes resistance to androgen-deprivation in prostate cancer. Am J Cancer Res.

[43] Wang H, Yan C, Shi X, Zheng J, Deng L, Yang L, Yu F, Yang Y, Shao Y (2015). MicroRNA-575 targets BLID to promote growth and invasion of non-small cell lung cancer cells. FEBS Lett.

[44] Wang Y-n, Xu F, Zhang P, Wang P, Wei Y-n, Wu C (2019). Cheng S-j: MicroRNA-575 regulates development of gastric cancer by targeting PTEN. Biomed Pharmacotherapy.

[45] Qin Y, Mi W, Huang C, Li J, Zhang Y, Fu Y (2020). Downregulation of miR-575 inhibits the tumorigenesis of gallbladder cancer via targeting p27 Kip1. OncoTargets therapy.

[46] Morris MR, Ricketts C, Gentle D, Abdulrahman M, Clarke N, Brown M, Kishida T, Yao M, Latif F, Maher ER (2010). Identification of candidate tumour suppressor genes frequently methylated in renal cell carcinoma. Oncogene.

[47] Kong R, Liu H, Shi Y, Man Q, Liu S (2021). COL14A1 promotes self-renewal of human liver cancer stem cells through activation of ERK signaling. J Bio-X Res.

[48] Hou L, Chen M, Yang H, Xing T, Li J, Li G, Zhang L, Deng S, Hu J, Zhao X (2016). MiR-940 inhibited cell growth and migration in triple-negative breast cancer. Med Sci monitor: Int Med J experimental Clin Res.

[49] Urh K, Žlajpah M, Zidar N, Boštjančič E (2021). Identification and validation of new cancer stem cell-related genes and their regulatory microRNAs in colorectal cancerogenesis. Biomedicines.

[50] Li H-t, Zhang H, Chen Y, Liu X-f, Qian J (2015). MiR-423-3p enhances cell growth through inhibition of p21Cip1/Waf1 in colorectal cancer. Cell Physiol Biochem.

[51] Guan G, Zhang D, Zheng Y, Wen L, Yu D, Lu Y, Zhao Y (2014). microRNA-423-3p promotes tumor progression via modulation of AdipoR2 in laryngeal carcinoma. Int J Clin experimental Pathol.

[52] Kuo W-T, Yu S-Y, Li S-C, Lam H-C, Chang H-T, Chen W-S, Yeh C-Y, Hung S-F, Liu T-C, Wu T (2016). MicroRNA-324 in human cancer: mir-324-5p and mir-324-3p have distinct biological functions in human cancer. Anticancer Res.

[53] Sun G-L, Li Z, Wang W-Z, Chen Z, Zhang L, Li Q, Wei S, Li B-W, Xu J-H, Chen L (2018). Mir-324-3p promotes gastric cancer development by activating Smad4-mediated Wnt/beta-catenin signaling pathway. J Gastroenterol.

