# Supplementary material for: Potential miRNA-gene interactions determining progression of various ATLL cancer subtypes after infection by HTLV-1 oncovirus
Source: BMC Med Genomics. 2023 Mar 28;16:62. doi: 10.1186/s12920-023-01492-0 (PMC10045051; doi:10.1186/s12920-023-01492-0)
Supplement: Supplementary file 1 — Supplementary Material 1 [file 12920_2023_1492_MOESM1_ESM.docx]

**Captions for supplementary data files**

**Supplementary data file 1:** *Z_summary_* and *medianRank* scores for each constructed weighted-gene co-expression network.

**Supplementary data file 2:** The unique genes in the specific and unique modules of ATLL (U_modules).

**Supplementary data file 3:** The identified unique DEGs for each ATLL subtype and DEMs.

**Supplementary data file 4:** Common genes between unique DEGs/DEMs and the genes/miRNAs in the specific modules (U_genes/U_miRNAs).

**Supplementary data file 5:** The experimentally validated target genes of U_miRNAs.
